# Supplementary material for: Understanding the Effect of Triazole on Crosslinked PPO–SEBS-Based Anion Exchange Membranes for Water Electrolysis
Source: Polymers (Basel). 2023 Mar 31;15(7):1736. doi: 10.3390/polym15071736 (PMC10098533; doi:10.3390/polym15071736)
Supplement: Supplementary file 1 [file polymers-15-01736-s001.zip › polymers-2279377-supplementary.pdf]

Supplementary Materials

Understanding the Effect of Triazole on Crosslinked PPO-SEBS-based Anion Exchange Membranes for Water Electrolysis

Jiyong Choi [1,2], Kyungwhan Min [1,2], Yong-Hwan Mo [3], Sang-Beom Han [3], and Tae-Hyun Kim [1,2,*]

[1] Organic Material Synthesis Laboratory, Department of Chemistry, Incheon National University, Incheon, 22012, Republic of Korea
[2] Research Institute of Basic Sciences, Core Research Institute, Incheon National University, Incheon, 22012, Republic of Korea
[3] Boyaz Energy, 165 Gasandigital 2-ro, Geumcheon-gu, Seoul, 08504, Republic of Korea
* Correspondence: tkim@inu.ac.kr; Tel.: +82-32-8358232

This file includes:
Characterization and measurement
Scheme S1–S3
Figures S1–S3
Table S1

Characterization and measurement

The chemical structure of the PPO and SEBS and their derivatives were identified via ^{1}H nuclear magnetic resonance (NMR) and Fourier transform infrared (FTIR) spectroscopy. The ^{1}H NMR spectra were obtained using a 400 MHz NMR spectrometer (Agilent 400-MR) using $CDCl_3$ as a reference. ATR-FTIR spectra were recorded on a PerkinElmer FTIR Spectrum Two.

Membrane thickness was determined with a micrometer (Mitutoyo Model 547-201, Japan). The hydroxide-ion conductivity (σ) of each membrane (size: 10 × 40 mm) was calculated by the equation:

$$\sigma = \frac{L}{R \times A}$$

where L is the distance between the reference electrodes and A is the cross-sectional length of the membrane. Alternating current (AC) two-point probe impedance spectroscopy was used to carry out ohmic resistance measurements (R). The electrode systems were connected at frequencies from 100 mHz to 2 MHz via an SI-1287 electrochemical interface and an SI-1260 overhead impedance/gain-phase analyzer. The conductivity measurements were performed from 20–80 °C at 20 °C steps, inclusive. For these conductivity measurements, the cell was immersed in DI water. The conductivity values were averaged over at least five trials of the same duration.

Using the back-titration method, the ion exchange capacity (IEC) value of each membrane was calculated. To neutralize the OH^- ions, the OH^- form membranes were stored for 24 h in a 0.01 M HCl standard solution. Residual HCl was back-titrated with a 0.01 M NaOH standard solution using a phenolphthalein indicator. The membranes were then dried to determine their weight (W_{dry}, g). The following equation was used to calculate the experimental IEC (in meq g^{-1}) as the moles of exchangeable hydroxide ions per gram:

$$\text{IEC (meq g}^{-1}) = \frac{(V_{0NaOH}C_{NaOH} - V_{xNaOH}C_{NaOH})}{W_{dry}},$$

The WU (%) and swelling ratio (SR, %) of each membrane were calculated by soaking the circular membranes in water at 20 °C and 80 °C. The membranes in their OH^- form were immersed in DI water for at least 24 h, the surface of the membrane was wiped with a tissue, the sample was quickly weighed (W_{wet}), and the length (l_{wet}) and thickness (t_{wet}) were quickly measured. Then, the membrane was dried under vacuum for 24 h, and the weight (W_{dry}), length (l_{dry}), and thickness (t_{dry}) of the dry membrane were also measured. The following equations were used to determine the WU (%) and SR (%):

$$\text{WU (\%)} = \frac{W_{wet} - W_{dry}}{W_{dry}} \times 100$$

$$\text{SR (\%)} = \frac{l_{wet} - l_{dry}}{l_{dry}} \times 100 \text{ or } \frac{t_{wet} - t_{dry}}{t_{dry}} \times 100$$

The gel fraction of the crosslinked membrane was measured by immersion in $CHCl_3$ at room temperature for 48 h. The membrane was then dried at 40 °C for 24 h. Subsequently, the gel fraction was determined by the following equation:

$$\text{Gel fraction (\%)} = \frac{W_a}{W_0} \times 100$$

where W_0 is the weight of the membrane before the gel fraction test, and W_a is the membrane weight after the gel fraction test.

A benchtop tensile tester (Shimadzu EZ-TEST E2-L) was used to measure the mechanical properties of the membranes at a crosshead speed of 1 mm min^{-1} at 25 °C under 50% relative humidity. The cross-sectional area of the initial sample was used to determine the engineering stress. The initial slope of the stress–strain curve was used to calculate Young's modulus (E). For this test, membrane samples were prepared in dumbbell shapes of 40 × 10 mm total area and 20 × 10 mm test area.

The thermal stability of the membrane was investigated by thermogravimetric analysis (TGA) using a Scinco TGA N-1000 instrument. The TGA was conducted at a heating rate of 10 °C min^{-1} from 50 to 800 °C under a nitrogen atmosphere.

Differential scanning calorimetry (DSC) measurements were performed using a PerkinElmer DSC 4000 to calculate the glass-transition temperature (T_g). Samples were prepared in aluminum pans and measured from −40 to 200 °C for two cycles at a heating and cooling rate of 10 °C min^{-1}. The second heating plot was used to determine T_g.

Scanning electron microscopy (SEM) was performed on a JEOL JSM-7800F instrument with a 15.0 kV accelerating voltage and an upper secondary electron detector to observe the surface properties. Samples were coated with Pt before imaging. The membranes were dried well in a vacuum oven at 80 °C before measurement.

The microphase separation of the membranes was observed using atomic force microscopy (AFM, Bruker MULTIMODE-8-AM, Billerica, MA, USA).

A Rigaku HR-XRD Smartlab diffractometer was used to collect the X-ray diffraction (XRD) spectra of the dried membranes. Small-angle X-ray diffraction scattering (SAXS) was measured at a scanning rate of 0.25° min^{-1} in a 2θ range from 0° to 6° with a Cu-Kα X-ray source (λ = 1.54 Å). The membranes were dried at room temperature prior to measurement.

Contact angles were measured statically on surface electro-optics (SEO) instruments and calculated using SEO Surfaceware-9. A membrane was placed on a flat surface to determine the contact angle. Subsequently, a 7-μL water droplet was placed on the surface of the membrane. A picture was taken 10 seconds after the water droplet touched the surface to obtain the contact angle. All measurements were performed at least three times, and the average values and the standard deviations were calculated and reported.

The bound water (non-freezing water) and free water (freezing water) contents of the membranes were determined by measuring the DSC of a membrane in a fully hydrated state with a PerkinElmer

DSC 4000. A fully hydrated membrane was sealed in an aluminum sample pan, and an empty sample pan was prepared as a reference. The sealed and empty pans were weighed. The sealed pan was frozen inside the DSC chamber to −40 °C as soon as possible after weighing, and that temperature was maintained for system equilibration. Then, the DSC chamber was heated to 20 °C with a heating rate of 2 °C min^{-1}. The amount of freezing water in each membrane was calculated by integrating the peak area of the melt endotherm. The degree of crystallinity of the water was obtained from the standard heat of fusion of pure water (334 J g^{-1}). The weight difference between the wet and dry membranes was used to determine the total water content. The freezing water and bound water contents were calculated using the following equations:

$$\text{Freezing water (\%)} = \frac{Melting\ enthalpy\ (Jg^{-1})}{Melting\ endothermic\ heat\ of\ fusion\ of\ pure\ water\ (334\,Jg^{-1})} \times 100$$

$$\text{Non} - \text{freezing bound water (\%)} = \text{total water content (\%)} - \text{freezing water content (\%)}$$

The OH^- form membranes were soaked in 1 M KOH solution at 80 °C for at most 960 h to evaluate the chemical stability of membranes. Before the measurements, each membrane was washed several times with DI water, and the free KOH inside the membrane was removed by soaking it in DI water for at least 24 h at room temperature. The ionic conductivity of each membrane was measured in DI water at 20 °C.

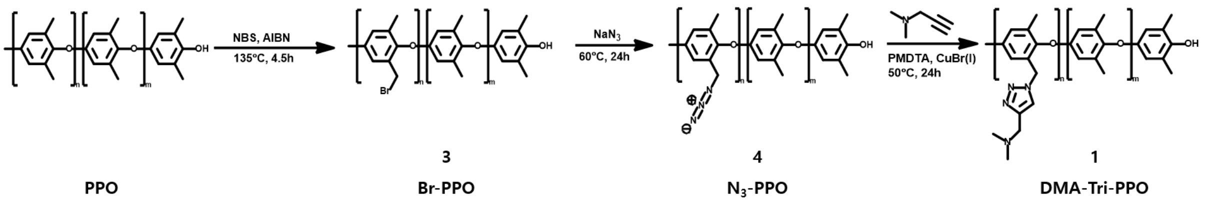

Scheme 1. Synthetic route to DMA-Tri-PPO **1.**

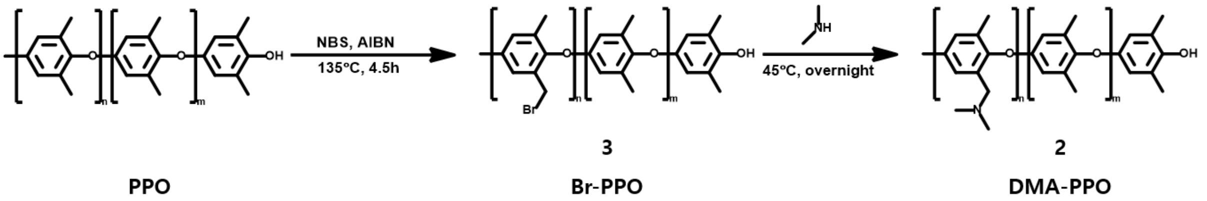

Scheme 2. Synthetic route to DMA-PPO **2.**

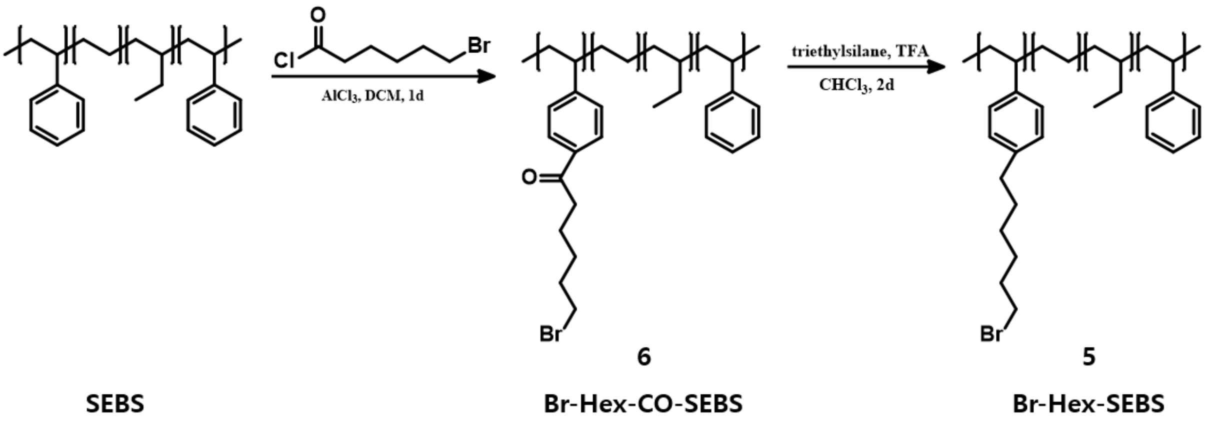

Scheme 3. Synthesis of Br-Hex-SEBS **5.**

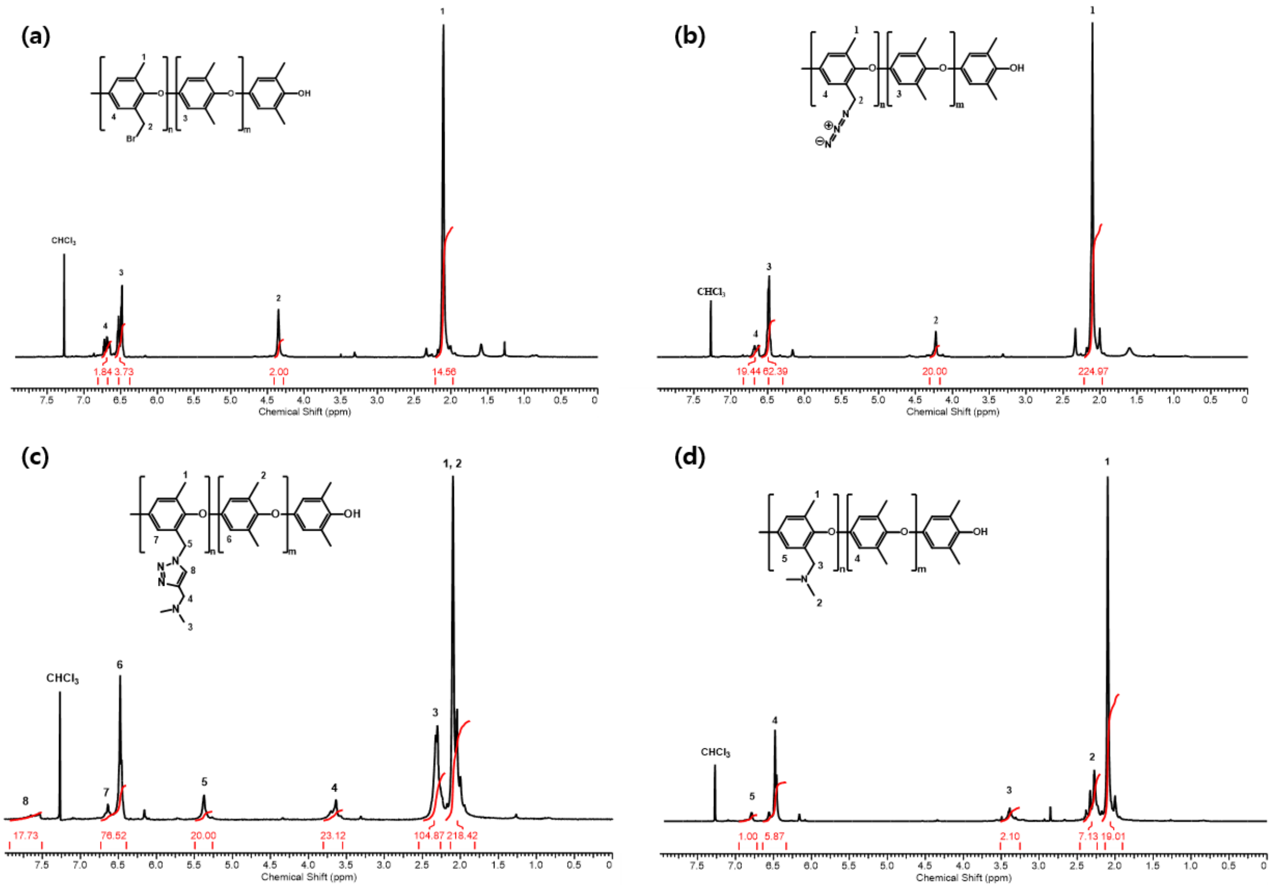

Figure S1. ^{1}H NMR spectra of (a) Br-PPO **3**, (b) N_3-PPO **4**, (c) DMA-Tri-PPO **1**, and (d) DMA-PPO **2.**

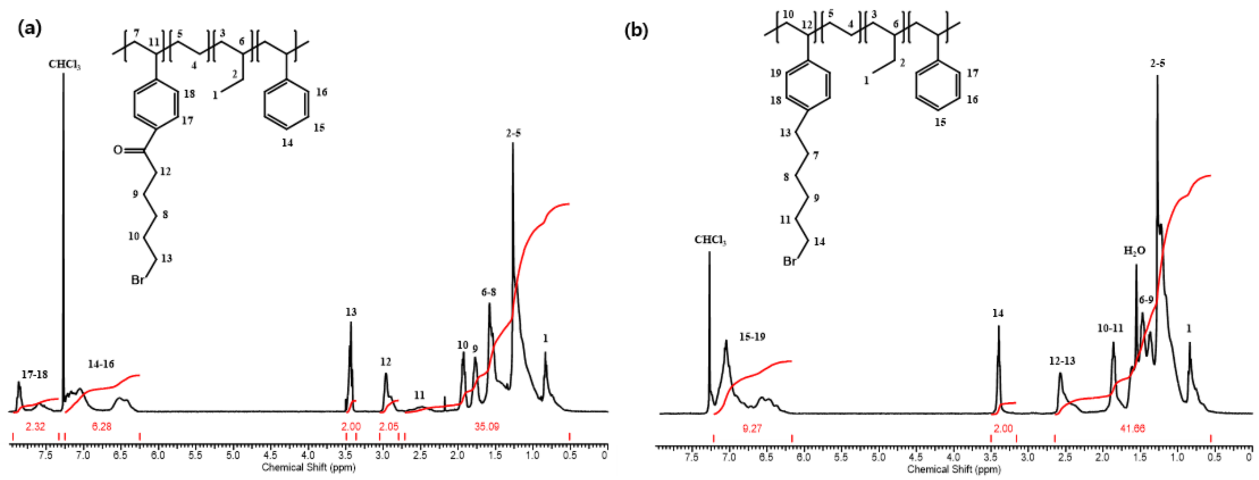

Figure S2. ^{1}H NMR spectra of Br-Hex-CO-SEBS **6** (a) and Br-Hex-SEBS **5** (b).

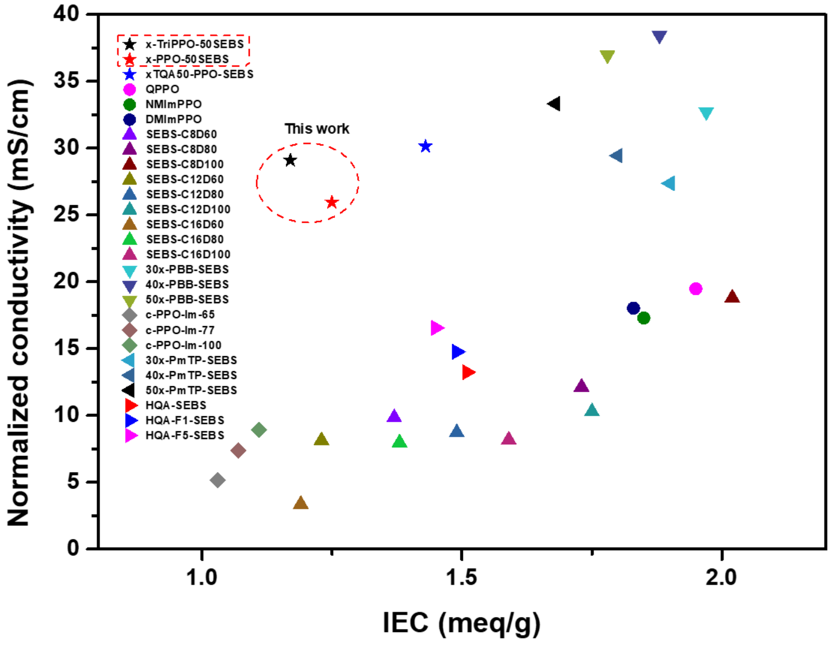

Figure S3. Normalized conductivity vs. IEC at 20 °C of both PPO-SEBS membranes compared with SEBS- and PPO-based AEMs [1–7].

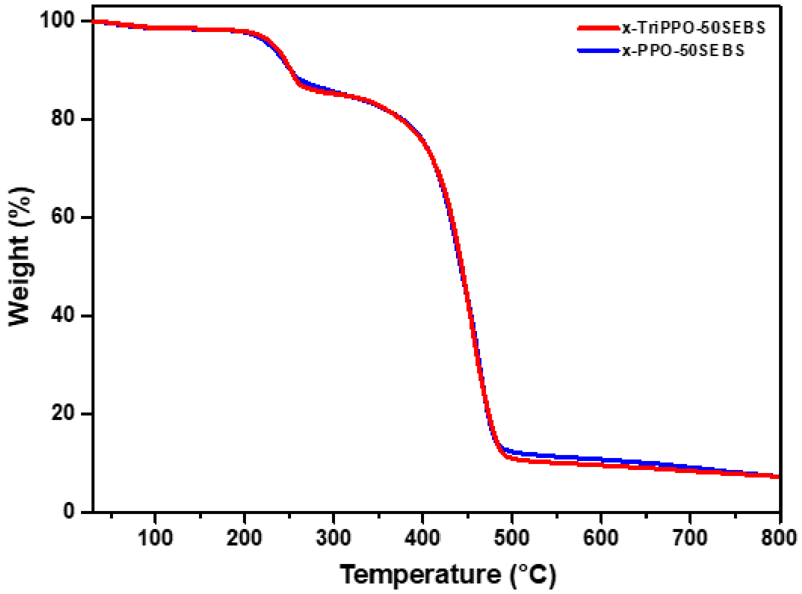

Figure S4. TGA graphs of the crosslinked PPO-SEBS membranes.

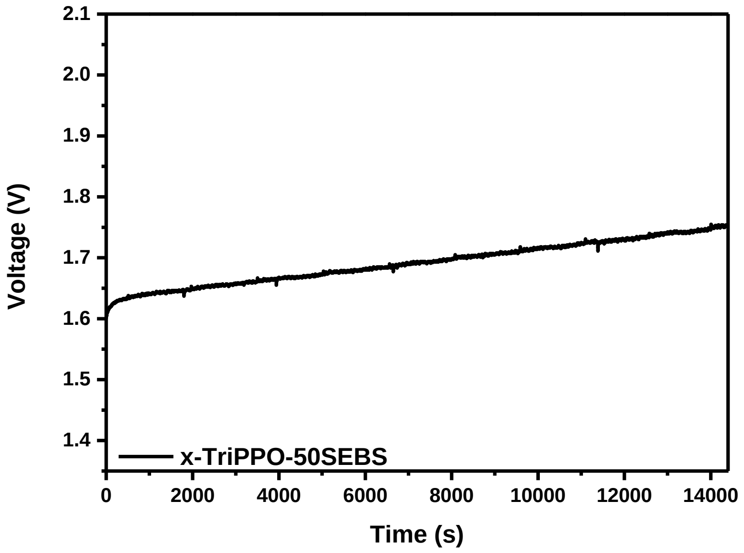

Figure S5. The durability of x-TriPPO-50SEBS membrane-based WE cell under a current density of 200 mA cm^{-2} at 70 °C.

Table S1. Gel fraction of the crosslinked PPO-SEBS membranes measured in $CHCl_3$ at room temperature.

Membrane	Gel fraction (%)
x-TriPPO-50SEBS	100
x-PPO-50SEBS	99.1

Table S2. Comparison of AEMs developed in this work to other AEMs [8–15].

AEM	A/C ionomer	Anode catalyst<br>Cathode catalyst	A/C electrolyte	Temp	AEMWE current density
x-TriPPO-50SEBS	FAA-3	4.0 mg/cm^2 IrO_2<br>0.5 mg/cm^2 Pt/C	1M KOH	70°C	0.71 A/cm^2@1.8V
x-PPO-50SEBS	FAA-3	4.0 mg/cm^2 IrO_2<br>0.5 mg/cm^2 Pt/C	1M KOH	70°C	0.42 A/cm^2@1.8V
HTMA-DAPP	TMA-70	3 mg/cm^2 Ni-Fe<br>0.5 mg/cm^2 PtRu/C	1M NaOH	85°C	5.5 A/cm^2@1.85V
HTMA-DAPP	HTMA-DAPP	2 mg/cm^2 IrO_2<br>0.4 mg/cm^2 PtRu/C	H_2O	60°C	0.4 A/cm^2@2.0V
PTFE-Sustainion®	Nafion	Ni-Mo<br>Fe-Ni-Mo	1 M KOH	80°C	1.0 A/cm^2@1.57V
PTP-90	NG	IrO_2<br>Pt/C	1M NaOH	75°C	1.0 A/cm^2@1.8V
PIS	Nafion	2 mg/cm^2 IrO_2<br>0.5 mg/cm^2 Pt/C	0.5 M KOH	80°C	0.55 A/cm^2@2.0V
x-QAPS	x-QAPS	Ni-Fe<br>Ni-Mo	H_2O	70°C	0.24 A/cm^2@1.8V
FAA-3-50	NG	1.8 mg/cm^2 $NiFe_2O_4$<br>2.7 mg/cm^2 NiFeCo	1 M KOH	60°C	0.24 A/cm^2@1.8V
Fumapem-3-PE-30	Fumion FAA-3	3 mg/cm^2 Iridium black<br>1 mg/cm^2 Pt/C	1M KOH	50°C	1.00 A/cm^2@1.8V

References

1. Zhu, Y.; He, Y.; Ge, X.; Liang, X.; Shehzad, M.A.; Hu, M.; Liu, Y.; Wu, L.; Xu, T. A benzyltetramethylimidazolium-based membrane with exceptional alkaline stability in fuel cells: role of its structure in alkaline stability. *J. Mater. Chem. A* **2018**, *6*, 527–534. https://doi.org/10.1039/C7TA09095A.
2. Sang, J.; Yang, L.; Li, Z.; Wang, F.; Wang, Z.; Zhu, H. Comb-shaped SEBS-based anion exchange membranes with obvious microphase separation morphology. Electrochimica Acta 2022, 403, 139500. https://doi.org/10.1016/j.electacta.2021.139500.
3. Min, K.; Chae, J.E.; Lee, Y.; Kim, H.-J.; Kim, T.-H. Crosslinked poly(m-terphenyl N-methyl piperidinium)-SEBS membranes with aryl-ether free and kinked backbones as highly stable and conductive anion exchange membranes. *Journal of Membrane Science* **2022**, *653*, 120487, doi:https://doi.org/10.1016/j.memsci.2022.120487.
4. Al Munsur, A.Z.; Lee, J.; Chae, J.E.; Kim, H.-J.; Park, C.H.; Nam, S.Y.; Kim, T.-H. Hexyl quaternary ammonium- and fluorobenzoyl-grafted SEBS as hydrophilic–hydrophobic comb-type anion exchange membranes. *J. Membr. Sci.* **2022**, *643*, 120029. https://doi.org/10.1016/j.memsci.2021.120029.
5. Min, K.; Lee, Y.; Choi, Y.; Kwon, O.J.; Kim, T.-H. High-performance anion exchange membranes achieved by crosslinking two aryl ether-free polymers: Poly(bibenzyl N-methyl piperidine) and SEBS. *J. Membr. Sci.* **2022**, *664*, 121071. https://doi.org/10.1016/j.memsci.2022.121071.
6. Zhang, X.; Cao, Y.; Zhang, M.; Wang, Y.; Tang, H.; Li, N. Olefin metathesis-crosslinked, bulky imidazolium-based anion exchange membranes with excellent base stability and mechanical properties. *J. Membr. Sci.* **2020**, *598*, 117793. https://doi.org/10.1016/j.memsci.2019.117793.
7. Sung, S.; Chae, J.E.; Min, K.; Kim, H.-J.; Nam, S.Y.; Kim, T.-H. Preparation of crosslinker-free anion exchange membranes with excellent physicochemical and electrochemical properties based on crosslinked PPO-SEBS. *J. Mater. Chem. A* **2021**, *9*, 1062–1079. https://doi.org/10.1039/D0TA10194J.
8. Liu, J.; Kang, Z.; Li, D.; Pak, M.; Alia, S.M.; Fujimoto, C.; Bender, G.; Kim, Y.S.; Weber, A.Z. Elucidating the Role of Hydroxide Electrolyte on Anion-Exchange-Membrane Water Electrolyzer Performance. *J. Electrochem. Soc.* **2021**, *168*, 054522. https://doi.org/10.1149/1945-7111/ac0019.
9. Chen, P.; Hu, X. High-Efficiency Anion Exchange Membrane Water Electrolysis Employing Non-Noble Metal Catalysts. *Adv. Energy Mater.* **2020**, *10*, 2002285. https://doi.org/10.1002/aenm.202002285.
10. Hu, X.; Huang, Y.; Liu, L.; Ju, Q.; Zhou, X.; Qiao, X.; Zheng, Z.; Li, N. Piperidinium functionalized aryl ether-free polyaromatics as anion exchange membrane for water electrolysers: Performance and durability. *J. Membr. Sci.* **2021**, *621*, 118964. https://doi.org/10.1016/j.memsci.2020.118964.
11. Li, D.; Park, E.J.; Zhu, W.; Shi, Q.; Zhou, Y.; Tian, H.; Lin, Y.; Serov, A.; Zulevi, B.; Baca, E.D.; et al. Highly quaternized polystyrene ionomers for high performance anion exchange membrane water electrolysers. *Nat. Energy* **2020**, *5*, 378–385. https://doi.org/10.1038/s41560-020-0577-x.
12. Park, H.J.; Lee, S.Y.; Lee, T.K.; Kim, H.-J.; Lee, Y.M. N3-butyl imidazolium-based anion exchange membranes blended with Poly(vinyl alcohol) for alkaline water electrolysis. *J. Membr. Sci.* **2020**, *611*, 118355, https://doi.org/10.1016/j.memsci.2020.118355.
13. Xiao, L.; Zhang, S.; Pan, J.; Yang, C.; He, M.; Zhuang, L.; Lu, J. First implementation of alkaline polymer electrolyte water electrolysis working only with pure water. *Energy Environ. Sci.* **2012**, *5*, 7869–7871. https://doi.org/10.1039/C2EE22146B.
14. Motealleh, B.; Liu, Z.; Masel, R.I.; Sculley, J.P.; Richard Ni, Z.; Meroueh, L. Next-generation anion exchange membrane water electrolyzers operating for commercially relevant lifetimes. *Int. J. Hydrog. Energy* **2021**, *46*, 3379–3386. https://doi.org/10.1016/j.ijhydene.2020.10.244.
15. Faid, A.Y.; Barnett, A.O.; Seland, F.; Sunde, S. Ternary NiCoFe nanosheets for oxygen evolution in anion exchange membrane water electrolysis. *Int. J. Hydrog. Energy* **2022**, *47*, 23483–23497. https://doi.org/10.1016/j.ijhydene.2022.05.143.
